# Role of laparoscopy in patients with peritoneal metastases considered for cytoreductive surgery and hyperthermic intraperitoneal chemotherapy (HIPEC)

**DOI:** 10.1186/1477-7819-12-270

**Published:** 2014-08-21

**Authors:** Thejus T Jayakrishnan, Anthony J Zacharias, Avishkar Sharma, Sam G Pappas, T Clark Gamblin, Kiran K Turaga

**Affiliations:** Division of Surgical Oncology, Department of Surgery, Medical College of Wisconsin, 9200 W Wisconsin Ave, Milwaukee, WI 53226 USA; Division of Surgical Oncology, Department of Surgery, Loyola University Medical Center, 2160 South 1st Avenue, Maywood, IL USA

**Keywords:** Peritoneal neoplasms, regional perfusion, chemotherapy, postoperative complications, surgical procedures, HIPEC, staging, laparoscopy

## Abstract

**Background:**

We hypothesized that diagnostic laparoscopy (DL) was feasible for the evaluation of patients with peritoneal carcinomatosis (PC) undergoing cytoreductive surgery and hyperthermic intraperitoneal chemotherapy (CRS + HIPEC).

**Methods:**

A retrospective review of PC patients treated from January 2010 to April 2013 was conducted. Data on tumor characteristics, treatment details and survival outcomes were extracted and analyzed.

**Results:**

Of the 101 PC patients (mean age 52.9 ± 14.1 years), 73 diagnostic laparoscopies DL (61 concurrent with CRS + HIPEC) were performed in 70 patients whereas 31 patients underwent direct exploratory laparotomy (EL). Complete laparoscopic assessment was possible in 63 cases (86.3%), resulting in 18 exclusions (27.7%) while 10 cases were converted to open due to inadequate laparoscopic visualization. Subsequently, CRS + HIPEC was performed in 85.4% (of 55 selected for HIPEC, DL) versus 74.2% (EL, *P* value = 0.20). Among those excluded from HIPEC at the initial operation, delayed HIPEC after conversion chemotherapy was achieved in 6 (of 11 with extensive disease, DL). The incidence of grade 3 to 5 complications was 0% DL versus 10% EL (*P* value = 0.2). There were no port site recurrences at mean follow up of 9.1 ± 8 months.

**Conclusions:**

Laparoscopy is a feasible technique for selecting patients with PC for CRS + HIPEC, and can help select patients for conversion chemotherapy in the setting of high peritoneal carcinomatosis index (PCI) score.

## Background

Peritoneal carcinomatosis (PC) represents an advanced stage of many cancers and is associated with poor survival rates [1–5]. Cytoreductive surgery (CRS) with hyperthermic intraperitoneal chemotherapy (HIPEC) was introduced in an attempt to improve the prognosis of these patients and incorporates surgical removal of all visible disease followed by chemical destruction of microscopic disease through chemoperfusion [6].

The most validated predictors of outcome in patients undergoing CRS + HIPEC are preoperative tumor burden measured in terms of the peritoneal carcinomatosis index (PCI) and completeness of cytoreduction (CC) [7–10]. Patients undergoing CRS + HIPEC in which surgery achieved complete or near complete cytoreduction (CC-0 or CCR-1) were shown to have significantly greater survival benefit over those who did not [7, 9, 11]. Survival analyses studies have shown that patients with PCI scores less than 19 (colorectal) and 10 (gastric) benefit most from CRS + HIPEC, facilitating the use of these scores as general cut-offs for excluding patients from this morbid procedure [12–15]. Exclusion of patients from definitive resection at laparotomy (a non-therapeutic laparotomy) is undesirable and may impede the timely enrollment of patients into alternate therapies [16]. Therefore, the role of preoperative imaging in CRS + HIPEC is to filter out patients with very high volume disease or no carcinomatosis. Despite advancements in cross-sectional imaging, the proportion of patients excluded at direct exploratory laparotomy for CRS + HIPEC is high (20 to 40%) [17, 18].

Diagnostic laparoscopy prior to resection is widely used in hepatopancreaticobiliary and colorectal cancer and has been shown to be effective in excluding unnecessary laparotomy associated with higher morbidity [19, 20]. The laparoscopy could be done concomitantly at the time of planned resection or as a separate staging procedure [19]. In addition, this may permit earlier enrollment of these patients into palliative or neoadjuvant therapy with potential impact on survival [16]. However, there is a paucity of data on the use of laparoscopy in the evolving field of cytoreductive surgery [21]. Since PC represents an advanced stage of cancer, visualization may be difficult due to adhesions from the cancer or prior surgical procedures. Additionally, there is potentially increased risk of complications such as visceral perforation in the setting of PC. The hypothesis for the present study was that laparoscopic evaluation is feasible for the evaluation of patients with peritoneal carcinomatosis selected for CRS + HIPEC.

## Methods

A retrospective review of patients treated for peritoneal carcinomatosis at the The Froedtert & the Medical College of Wisconsin’s Regional Cancer Therapy Program between January 2010 and April 2013 was performed after approval by the Institutional Review Board (IRB). Demographic information of patients who underwent laparoscopic/laparotomy evaluation for CRS + HIPEC was extracted. Patients who had multiple HIPECS as well as those who underwent CRS outside and were referred to our institute for further management were excluded from the study. Data on tumor characteristics, operative details, hospital course, and morbidity (Clavien-Dindo classification) and survival outcomes were extracted and entered into a pre-specified data extraction sheet. The length of the hospital stay was calculated from the date of the surgical procedure to the date of discharge. The follow-up information current with 4 April 2013 (date of IRB approval) was captured.

All patients referred for treatment under the program underwent routine preoperative computerized tomography (CT) imaging at the institute when recent outside scans were unavailable. If outside scans were available, these were reviewed by radiologists at the institute. Preoperative work-up also included all blood work-ups and relevant tumor markers. Patients selected for CRS + HIPEC by imaging routinely underwent laparoscopic staging prior to laparotomy. However, patients were selected for direct laparotomy if they had recent surgical evaluation prior to their referral to our program and the disease appeared amenable for CRS + HIPEC based on operative notes and cross sectional imaging. Patients were classified as extensive disease for exclusion from CRS + HIPEC for PCI scores higher than 11 for gastric cancer and 19 for colorectal and non-gastric primaries. In addition, patients with no suspicion for peritoneal carcinomatosis were also excluded from CRS + HIPEC.

### Technique for diagnostic laparoscopy

The site of first port placement during diagnostic laparoscopy (DL) was decided at the surgeon’s discretion based on imaging and clinical findings of the patient. The preferred technique for first creation of pneumoperitoneum was via the optical access technique in the left upper quadrant. In others, a Hasson’s technique was used to establish pneumoperitoneum. After ruling out significant adhesions at the anterior abdominal wall, systematic visual examination of the abdomen was performed to generate the PCI score (complete laparoscopy) [7, 22]. Systematic examination of the abdomen was then performed including special attention to peri-splenic, peri-hepatic, pelvic, omental bursa and bowel.

### Technique for hyperthermic intraperitoneal chemotherapy

After cytoreduction, HIPEC was performed using the closed technique and dosing of therapy was based on standard published consensus guidelines [23].

### Subsequent management

Patients excluded from CRS + HIPEC were either enrolled into a chemotherapy program with plan for future CRS + HIPEC or discharged to hospice or home care.

The first post-operative visit after CRS + HIPEC was planned at 6 weeks. Subsequent visits were planned to take place every 3 months for 2 years and subsequently, every 6 months for 3 years. The visits included physical examinations and lab chemistries including basic hematological parameters, blood chemistry, and tumor markers as appropriate. Imaging follow-up was performed using CT scans for the majority of the patients. Magnetic resonance imaging (MRI) and positron emission tomography (PET) scans were used in some patients, but were not routine. The most recent images were reviewed at every visit.

### Statistical analyses

Statistical calculations were performed with STATA software Version 12.1 (StataCorp, Texas, USA). Continuous data are summarized as mean and standard deviation. Comparison was performed using Student’s t-test for continuous data. Categorical variables were expressed as valid percentages and compared using the chi-squared test or Fisher’s exact test as appropriate. The survival analysis was performed using Kaplan-Meier plots with log-rank analyses. Alpha was fixed at 0.05 for statistical significance.

## Results

The baseline data and outcomes from CRS + HIPEC for the 101 patients studied are represented in Table 1. The most prevalent primary was appendiceal (47.5%), followed by colorectal (33.7%) and gastric (6.9%). The presentation of peritoneal carcinomatosis was synchronous in 22 (21.8%) and metachronous in 79 (78.2%) patients.

**Table 1:** **Baseline characteristics and outcomes of patients treated for peritoneal carcinomatosis (N = 101)**

Characteristic	Mean (SD)	Frequency n (%)
Age of primary diagnosis in years		
Mean	52.3 (13.4)	
Sex		
Male		49 (48.5)
Female		52 (51.5)
Primary Tumor Diagnosis		
Appendiceal		48 (47.5)
Colorectal		34 (33.7)
Gastric		7 (6.9)
Ovarian		3 (3.0)
Sarcoma		3 (3.0)
Others		6 (5.9)
Grade of primary tumor (n = 85)		
Grade 1 (Well differentiated)		36 (42.9)
Grade 2 (Moderately differentiated)		23 (27.4)
Grade 3 (Poorly differentiated/signet ring)		25 (29.8)
Preoperative imaging		
Computed tomography (CT) scan		89 (88.1)
Magnetic resonance imaging (MRI) scan		41 (40.6)
Positron emission tomography (PET) scan		62 (69.7)
Intraoperative PCI score (n = 74)	15.5 (11.7)	
HIPEC completed		73 (72.3)
Completeness of cytoreduction (n = 65)		
CC0		52 (80.0)
CC1		9 (13.9)
CC2		4 (6.2)
Length of stay	11.3 (9.1)	
Grade 3 to 5 complications		15 (20.5)
Chemotherapy received		46 (45.5)
Before CRS		16 (34.8)
After CRS		19 (41.3)
Both before and after CRS		11 (23.9)
Patients alive and under follow up		82 (81.2)
Status at last office visit (n = 82)		
No disease		31 (37.8)
Stable disease		24 (29.3)
Progressive disease		27 (32.9)

*CRS*, cytoreductive surgery; *HIPEC*, hyperthermic intraperitoneal chemotherapy; *PCI*, peritoneal carcinomatosis index.

Laparoscopic evaluation (DL) was performed 73 times (12 as a separate procedure and 61 concurrent with planned CRS + HIPEC) in 70 patients, whereas 31 patients were selected for direct exploratory laparotomy (EL). Primary tumor resection prior to CRS was performed in 78 patients (77.2%) overall: 56 (80.0%) in the DL group versus 22 (71.0%) receiving EL, *P* value = 0.318. The respective median intervals to second surgeries were 11.0 (interquartile range (IQR) 4.8 to 30.3) months for DL versus 19.8 (11.0 to 50.4) months for EL.

### Laparoscopy outcomes

**Figure 1:**
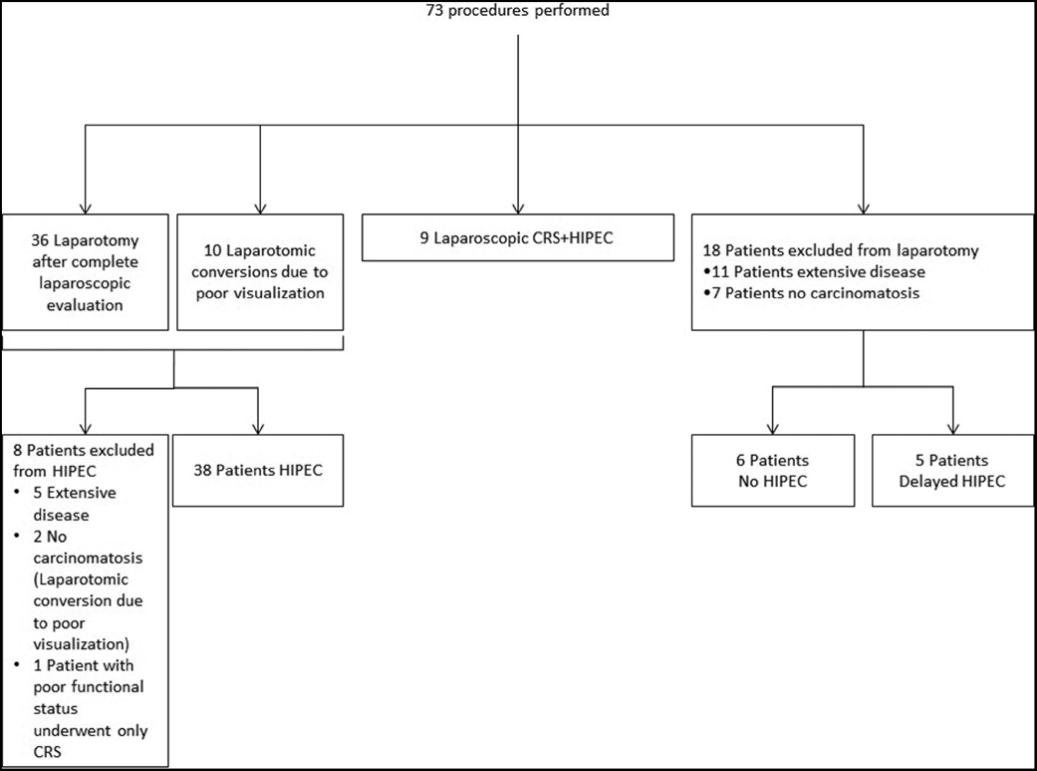
**Flowchart depicting the outcomes of diagnostic laparoscopy (DL).** CRS, cytoreductive surgery; HIPEC, hyperthermic intraperitoneal chemotherapy.

**Figure 2:**
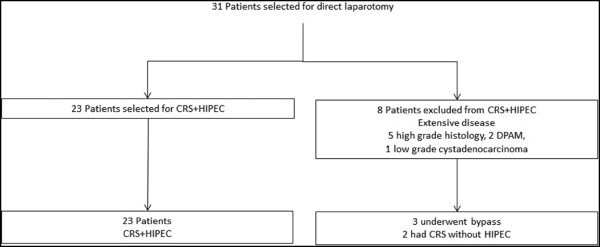
**Flowchart depicting the outcomes of patients who did not undergo diagnostic laparoscopy.** CRS, cytoreductive surgery; HIPEC, hyperthermic intraperitoneal chemotherapy; DPAM, disseminated peritoneal adenomucinosis.

The technical details and outcomes of the DL, stratified by the timing of the procedure, are described in Table 2 and represented in Figure 1. The DL entry technique was optical trocar in 68.5% and Hasson trocar in 30.1% of the procedures. The left subcostal region was the favored site of entry in 60.3%, while a midline periumbilical incision was used in 39.7% of the procedures. Complete laparoscopic evaluation was possible in 63 cases (of 73, 86.3%) with 18 (of 73, 27.7%) patients being excluded from laparotomy for CRS + HIPEC. The reason for exclusion was extensive disease (high PCI) in 11 cases and absence of carcinomatosis in 7. Laparoscopic CRS + HIPEC was performed in nine patients, with all patients achieving CC0 cytoreduction and successful completion of HIPEC. Of the 46 patients who underwent laparotomy (4 with a separate laparoscopy and 42 with a concurrent laparoscopy), 38 were selected for CRS + HIPEC. HIPEC was not performed in six patients with extensive disease (who underwent resection of primary and cytoreduction without HIPEC) and two patients with no carcinomatosis (cases that were inadequately visualized in laparoscopy due to adhesions, these were converted to open). Therefore, CRS + HIPEC was successfully performed in 85.4% (DL, 47 of 55 not excluded by laparoscopy) compared to 74.2% (23 of 31, *P* value = 0.20) in the EL group (Figure 2). The incidence of Clavien-Dindo grade 3 to 5 complications among those excluded from HIPEC were 0% with DL versus 10% with EL (*P* value = 0.2).

**Table 2:** **Technical details and outcomes of diagnostic laparoscopy (DL) stratified by the timing of the procedure with respect to planned laparotomy for cytoreductive surgery and hyperthermic intraperitoneal chemotherapy (CRS + HIPEC)** ^**a**^

Characteristic	Procedure done concurrently with planned laparotomy for CRS + HIPEC (n = 61)	Procedure done separately prior to laparotomy for CRS + HIPEC (N = 12)
Entrance technique		
Visual entry system	43 (70.5)	8
Hasson trocar	18 (29.5)	4
Site entry		
Left upper quadrant	38 (62.3)	6
Midline periumbilical	23 (37.7)	6
Laparoscopy converted to open (inadequate visualization due to adhesions)	10 (16.4)	0
Excluded from laparotomy for CRS + HIPEC after adequate evaluation	10 (19.6)	8
Reason for exclusion		
Extensive disease	5	6
No carcinomatosis	5	2
Completed HIPEC	44 (86.3)	3 (1 Patient underwent CRS only due to poor functional status)
Open HIPEC	35	3
Laparoscopic HIPEC	9	0
Excluded from HIPEC at laparotomy (n = 42: 32 cases selected after complete laparoscopic evaluation and 10 cases of laparoscopic conversion to open)	7	0
Reason for exclusion		
Extensive disease	5	
No carcinomatosis (Cases converted from laparoscopy due to inadequate evaluation)	2	
Delayed HIPEC for those excluded at laparoscopy	2	3

^a^Two patients underwent both concurrent and separate DL.

Of the 11 cases with extensive disease that were excluded from CRS + HIPEC by laparoscopy, 5 received delayed CRS + HIPEC following systemic chemotherapy and down-staging of the disease. At a mean follow-up of 9.1 ± 8 months, 82 patients were alive. At the latest office visit, 31(37.8%) had no disease while 24(29.3%) had stable disease. Progressive disease was detected in 27(32.9%) patients. There was no port-site recurrences at current follow-up.

## Discussion

CRS + HIPEC is an evolving treatment option for treatment of patients with peritoneal carcinomatosis and has been shown to significantly improve survival in multiple studies [11, 24–26]. The present study demonstrates the feasibility and safety of laparoscopy in the treatment paradigm of patients with suspected peritoneal disease.

The maximum impact of the laparoscopy is in reducing non-therapeutic laparotomies for patients with no disease (38.9%) and in assessing patients with high burden of disease with high grade histology (61.1%), allowing the disease to be down-staged with systemic chemotherapy. An important finding was that many patients with extensive disease who were excluded by DL were able to subsequently undergo CRS + HIPEC after the downgrading of their disease by chemotherapy (5 out 11 patients with extensive disease excluded by DL) (Figure 1).

Based on our institutional practice, a diagnostic laparoscopy was not routinely utilized in patients with low-grade disease such as DPAM unless concerns for completion of cytoreduction were present, or in patients with bowel obstruction and in patients with a hostile abdomen, which does constitute a significant proportion of patients in a practice at a peritoneal surface malignancy center.

Oncological concerns of a laparoscopy include incomplete assessment of the peritoneal cavity and port site recurrences. In our study, we found that we were able to assess the PCI score more as a threshold rather than for accuracy. We recognize that open assessment of the peritoneum may be superior to laparoscopy especially for the right hemidiaphragm, omental bursa and the pelvis, yet believe that an assessment of high burden of disease is easily feasible with a laparoscopy.

Patients selected for HIPEC often belong to advanced age groups associated with poor performance status resulting in increased morbidity rates [27, 28]. The peritoneal carcinomatosis index is a well-validated tool of prognostic significance for assessing the burden of peritoneal disease and is used for selecting patients for CRS + HIPEC [7]. The sensitivity of cross-sectional imaging to accurately describe peritoneal carcinomatosis remains low despite the recent advancements [16, 20, 29, 30]. As many as 20 to 40% of the patients considered eligible for HIPEC based on imaging are excluded from HIPEC at laparotomy [17, 18]. Such non-therapeutic laparotomies may adversely affect the outcome of this vulnerable population with short life period, as well as affecting the physician patient relationship, thereby supporting the use of a diagnostic laparoscopy.

The use of laparoscopy in PC has hitherto remained limited due to technical concerns that the adhesions from cancer and past surgeries may hinder adequate assessment and possibly result in an increased rate of complications [21, 31]. In the present study, DL was successful in a majority (89.2%) of the patients and was not associated with any complications.

All patients underwent advanced imaging (triple phase helical CT scans/MRI) routinely before the surgical evaluation for HIPEC. All outside images were also reviewed at the institute. Therefore the high rate of exclusion from CRS + HIPEC (28.4%) reflects the low fidelity of imaging for classifying patients for CRS + HIPEC. This is lower than the exclusion rates reported for gallbladder cancer and also for pancreatic cancer in the pre-neoadjuvant paradigm and could be attributed to the good quality cross-sectional imaging [16, 19, 20, 30, 32, 33]. The fact that many of these patients were referred from outside following incidental diagnosis for peritoneal carcinomatosis during surgery for primary tumor and had adequate EL evaluation may also have contributed to the lower rate of exclusion from curative surgery.

Use of laparoscopy for staging may permit its extension to performing HIPEC when feasible. In the group of patients evaluated for this study, CC0 cytoreduction followed by successful HIPEC was achieved laparoscopically in nine patients. Other groups have also reported the feasibility of laparoscopic HIPEC [21]. Since most of the patients would need to continue chemotherapy after HIPEC, a laparoscopic procedure may permit lowering of this convalescent period to chemotherapy with potential impact on the outcome.

The routine use of laparoscopy has significant financial implications as well since the laparoscopy adds to the overall cost of therapy. In a previous paper, we studied the cost effectiveness of laparoscopy in pancreatic cancer and demonstrated that laparoscopy was cost-effective at high probability for exclusion from resection [33]. This is directly related to pretest probability for exclusion as well as the sensitivity of laparoscopy. which may be subjective to the surgeon and influenced by the experience. The use of imaging modalities like diffusion-weighted MRI and PET scanning for the identification of peritoneal disease is evolving but may increase the cost differential dramatically. These modalities were used during diagnosis and follow-up of the patients, but the sensitivity of these modalities is yet to be validated. The radiological evaluation permits only anatomic characterization of the extent of the disease whereas laparoscopy permits pathological diagnosis as well with potential prognostic value.

A potential limitation of the study is that the sample was small in size and taken from a single institution. The study may suffer from confounding biases inherent to the observational study design. In addition, we did not formally correlate the laparoscopic PCI with an open PCI, although we believe that a laparoscopic PCI would under-stage rather than over-stage someone with disease and thus not alter the algorithms proposed.

## Conclusions

Laparoscopy is feasible in patients with peritoneal carcinomatosis who are expected to undergo cytoreductive surgery. It can filter out ineligible patients with either no carcinomatosis or extensive disease precluding complete cytoreduction during CRS + HIPEC. Technical and oncological concerns of performing a laparoscopy were underwhelming based on our study.

## Abbreviations

CC (0: 1,2): completeness of cytoreduction
CRS + HIPEC: cytoreductive surgery and hyperthermic intraperitoneal chemotherapy
CT: computerized tomography
DL: diagnostic laparoscopy
DPAM: disseminated peritoneal adenomucinosis
EL: exploratory laparotomy
IQR: interquartile range
MRI: magnetic resonance imaging
PC: peritoneal carcinomatosis
PCI: peritoneal carcinomatosis index
PET: positron emission tomography.
